# Non-adherence to cervical cancer screening recommendations among women in Eswatini: a cross-sectional study

**DOI:** 10.1186/s12889-023-15022-1

**Published:** 2023-02-08

**Authors:** Phinda G. Khumalo, Mariko Carey, Lisa Mackenzie, Rob Sanson-Fisher

**Affiliations:** 1 School of Medicine and Public Health, Health Behaviour Research Collaborative College of Health The University of Newcastle, Medicine, and Wellbeing, NSW 2308 Callaghan, Australia; 2 Hunter Medical Research Institute, New Lambton, NSW Australia; 3 Health Behaviour Research Collaborative, The University of Newcastle, Hunter Medical Research Institute, Lot 1, Kookaburra Cct, New Lambton Heights , NSW 2305 Australia; 4 School of Medicine and Public Health, Centre for Women’s Health Research College of Health The University of Newcastle, Medicine, and Wellbeing, NSW 2308 Callaghan, Australia

**Keywords:** Adherence, Uterine cervical neoplasms, Screening, Guidelines, Eswatini

## Abstract

**Background:**

In 2018, Eswatini had the world's highest age-standardised cervical cancer incidence rate. Cervical cancer screening reduces women’s risk of invasive cervical cancer. Data on adherence to cervical cancer screening recommendations in Eswatini are scarce. The purpose of the current study was to determine Eswatini women’s self-reported adherence to cervical cancer screening recommendations, attitudes toward screening, and factors associated with non-adherence.

**Methods:**

A cross-sectional survey of women (*n* = 377) aged 25 to 59 accessing primary healthcare clinics (*n* = 4) in Eswatini assessed screening participation, attitudes and knowledge regarding cervical cancer screening, and socio-demographic variables. Adjusted logistic regression was used to assess factors associated with non-adherence to Eswatini cervical cancer screening recommendations.

**Results:**

One hundred and sixty-six (44%) women were classified as adherent to cervical cancer screening recommendations. Attitudinal barriers endorsed by over one-third of participants included a perceived low risk of cervical cancer (*n* = 161, 43%) and a view that screening is likely to be painful (*n* = 146, 38%). Participants had higher odds of being classified as non-adherent if they: were single compared with married (OR = 1.78, 95% CI: 1.05, 3.01, *p* = 0.03), perceived screening as likely painful (OR = 4.43, 95% CI: 2.62, 7.46, *p* < 0.001); and had not been advised by a doctor/ nurse to screen (OR = 2.82, 95% CI: 1.71, 4.64, *p* < 0.001). Also, a 1-year increase in age was associated with an increase in the odds of being classified as non-adherent (OR = 1.42, 95% CI: 1.39, 1.45, *p* = 0.01).

**Conclusions:**

Self-reported adherence was moderate among this group of women. Tailored interventions are needed to increase participation in cervical cancer screening, especially for those women with characteristics associated with being classified as non-adherent. Primary healthcare clinic nurses (and other health providers) may contribute toward improving participation in cervical cancer screening by advising eligible women to screen and providing health education addressing negative attitudes toward screening.

## Background

### Cervical cancer is a major public health concern in Eswatini

Although cervical cancer incidence and mortality rates have drastically declined in developed countries, incidence and mortality estimates remain high in many developing countries, especially in sub-Saharan Africa [1]. In Eswatini, cervical cancer is the most common cancer among women, with an estimated incidence rate of 84.5 per 100 000 female population [2]. It is the leading cause of cancer-related death in women, with an estimated mortality rate of 55.7 per 100 000 female population [2].

### Eswatini cervical cancer screening recommendations

The Eswatini Standardised Cancer Guidelines of 2018 recommend that women initiate cervical cancer screening at the age of 25 or at the time of diagnosis of Human Immunodeficiency Virus (HIV) seropositivity. It is recommended that women exit screening at the age of 59 years after a recent negative screening test (if known or assumed to be “HIV-negative”). Cervical cancer screening intervals are tailored to a woman’s HIV and/or Human Papillomavirus (HPV) statuses. It is recommended that women screen: (i) at least once in three years if they are “HIV-negative” and “HPV-negative”, (ii) at least once in two years if they are “HIV-negative” and “HPV-positive” (or their status is unknown), and (iii) at least once in 12 months if they are “HIV-positive”. In Eswatini, both cytology (Papanicolaou [Pap] smear) and Visual Inspection with Acetic Acid (VIA) are available; however, VIA is the most widely used screening option for precancerous lesions [3].

### Improving adherence to cervical cancer screening is of critical importance to public health

Adherence to cervical cancer screening recommendations is one of the critical determinants of a cervical cancer screening program’s effectiveness and efficiency [4]. In high income countries, regular cervical cancer screening is estimated to reduce the incidence of cervical cancer and related mortality by 50–60% [5] and 41–92% [6], respectively. Modelling analysis conducted in 78 low-income and lower-middle-income countries (including 41 sub-Saharan countries) suggested that adding twice-lifetime screening resulted in a 96·7% [91.3–96.7] reduction in incidence [7]. Given the importance of adherence to cervical cancer screening recommendations, it is imperative to understand and address non-adherence in any cervical cancer screening program.

### Studies assessing adherence to cervical cancer screening recommendations in sub-Saharan Africa are sparse

While numerous sub-Saharan African studies have examined women’s participation in cervical cancer screening (history of ever screening), only a few studies have studied adherence to cervical cancer screening recommendations (regularity of screening). In a recent survey conducted in clinics in three of the four regions of Eswatini, 29.3% of women aged 18–69 years reported having previously received cervical cancer screening [8]. Another community-based study conducted in two regions of Eswatini revealed that, in 2017, 5.2% of women aged 30 to 65 had ever been screened [9]. Both studies only assessed the history of ever screening for cervical cancer without considering the regularity of screening or adherence to cervical cancer screening recommendations. To our knowledge, no previous studies explored Swati women’s adherence to cervical cancer screening recommendations.

### Attitudes towards cervical cancer screening

Previous studies in sub-Saharan Africa have considered women’s attitudes toward cervical cancer screening related to willingness to screen, availability of screening, availability of partner or health provider support, preference for health provider based on age and sex, perception of cervical cancer personal risk, seriousness or severity of cervical cancer, the fatality of cervical cancer, fear associated with screening, pain associated with screening, affordability of cervical cancer screening, and embarrassment related to screening [10–14]. Attitudes towards cervical cancer screening have not been evaluated comprehensively in Eswatini. In a previous study conducted in one region of Eswatini, most participants believed that: screening helped prevent cervical cancer, screening was harmless, and screening was not expensive [15]. Another study using one item to assess attitude toward cervical cancer screening reported that 61% of participants agreed that all women should be screened for cervical cancer [9].

Most sub-Saharan African research investigating the association between attitudes and participation in cervical cancer screening has considered attitudes as a binary variable categorised as favourable and unfavourable or positive and negative [13, 16–18]. A small proportion of this research has reviewed the association between individual attitude items and participation in cervical cancer screening [9, 14]. Therefore, further research into the specific attitudes and beliefs associated with participation in cervical cancer screening is needed so that interventions may target specific attitudes.

### Factors associated with non-adherence to cervical cancer screening recommendations

Aiming to tackle negative attitudes towards cervical cancer screening may be partially effective but unlikely to fully address the problem of poor participation without considering other factors that may influence participation [19]. Though limited, sub-Saharan African studies have shown associations between non-adherence and factors such as socio-demographic factors, the role of healthcare providers, financial factors, and cervical cancer knowledge. Non-adherence tends to be associated with older age [20], lower educational attainment [21], being single [22], lack of health professional advice [23], low socioeconomic status [24], and lack of knowledge about cervical cancer and its prevention [18]. Similarly, poor healthcare-seeking behaviour, long-distance and time to health facility [25], and not having health insurance [26] seem to be associated with non-adherence to cervical cancer screening recommendations.

No study has assessed both attitudes and socio-demographic factors associated with non-adherence to Eswatini cervical cancer screening recommendations. Findings from previous sub-Saharan African research may not generalise to the Eswatini context due to the diverse culture, religion, societal organisation, health organisation, and levels of development in Sub-Saharan Africa [27, 28]. Exploring factors associated with non-adherence to screening guidelines in Eswatini will have important implications for policy and practice to improve adherence and reduce cervical cancer morbidity and mortality. In order to extend what is known about adherence, attitudes and socio-demographic factors associated with non-adherence to cervical cancer screening recommendations in sub-Saharan Africa, we conducted the current study to provide data about Eswatini. Our aims were to explore, amongst a sample of Eswatini women, 1) self-reported adherence to cervical cancer screening recommendations, 2) attitudes toward cervical cancer screening and 3) factors associated with non-adherence to cervical cancer screening recommendations.

## Methods

### Design and setting

A cross-sectional study was conducted in four primary healthcare clinics in Eswatini. A total of 94 primary healthcare clinics were eligible (i.e., provided care to at least 50 women aged 25 to 59 years per week at the time of data collection [October to December 2021]). The 94 clinics were stratified by the four geographic regions of Eswatini (Hhohho, Lubombo, Manzini, and Shiselweni), and one clinic was selected from each region using convenience sampling.

### Sample

Women who: (i) attended one of the four participating clinics during the data collection period, (ii) were aged between 25 and 59 years, and (iii) had no history of cervical cancer or hysterectomy were invited to participate in the study.

### Recruitment

Eligible women were approached by a research assistant in the clinic waiting room and invited to participate in the study. Those who agreed to participate were asked to sign a consent form (available in English and siSwati). The ages of both consenters and non-consenters were documented in a study log sheet.

### Survey development

Previous research [9, 29–33], World Health Organisation guidelines for screening and treatment of precancerous lesions for cervical cancer prevention [34], and Eswatini standardised cancer guidelines [3] informed the survey items. Survey items were reviewed by six health behaviour experts and revised accordingly. The survey was piloted in one clinic among 25 women and modified according to their feedback.

### Data collection

A self-administered pen‐and‐paper survey was given to each participant to complete while they awaited their turn to be seen by the nurse or at the end of their clinic visit. Survey completion took approximately 15 min.

### Measures

Self-reported screening participation: A multiple-choice question inquiring about the timing of the last visual inspection of the cervix with acetic acid (VIA) screening test was asked to assess screening participation. A question stem “have you ever had a visual inspection of the cervix with acetic acid (VIA) screening test for cervical cancer?” preceded the following responses (i) yes, in the past 12 months, (ii) yes, in the past 13 to 24 months ago, (iii) yes, in the past 25 to 36 months, (iv) yes, more than 36 months ago, and (v) never. A similar method of assessing adherence was used in Mozambique [35]. In the current study, participant recall was improved by ensuring respondents understood the questions (i.e., providing detailed explanations of what the VIA screening test entails within the paper survey).

Attitudes toward cervical cancer screening: Attitudes were assessed using 11 4-point Likert Scale items with strongly agree, agree, disagree, and strongly disagree as response options. Eight of these items assessed negative attitudes, while three assessed positive attitudes. Potential attitudes were derived from previous cervical cancer screening studies [30, 32].

Knowledge regarding cervical cancer screening: Knowledge items were derived from previous studies. Ten multiple-choice items were used to assess the knowledge regarding cervical cancer risk factors [29, 32, 36]. Six multiple-choice questions were used to assess knowledge about the Eswatini recommended screening intervals [3, 34]. Four items with true/false response options were used to measure knowledge regarding the benefits of screening [31, 33, 37]. In addition, three true/false items were used to evaluate knowledge concerning the meaning of screening results [33]. Each knowledge item was scored 1 if correct and 0 if incorrect.

#### Socio-demographic variables

We collected data on age, marital status, level of education, and availability of electricity in the participant’s household [used as a proxy to estimate the participant’s socioeconomic status] [9].

#### Cervical cancer screening accessibility-related characteristics

Accessibility was measured using two items (clinic visits in the past six months and travel time to the clinic). These items were derived from previous research [38, 39].

#### Health status

Two multiple-choice items (with yes/no/I don’t know responses) were used to assess participants’ human immunodeficiency virus (HIV) and human papillomavirus (HPV) statuses.

### Statistical analysis

Age distributions of consenters and non-consenters were compared using a Pearson’s Chi-squared test to assess selection bias. A dichotomous variable (adherent vs non-adherent) was created from the self-reported screening participation item [35]. Based on the Eswatini standardised cancer guidelines [3], women were regarded as adherent if they met at least one of the following conditions: (i) self-reported being “HIV-positive” and screened in the past 12 months, (ii) self-reported being “HIV-negative” and “HPV-positive” and screened in the past 13 to 24 months, and (iii) self-reported being “HIV-negative” and “HPV-negative” and screened in the past 25 to 36 months. An additional dichotomous variable describing the history of ever-screening (with responses 0—no and 1—yes) was created from the self-reported screening participation item. Both histories of ever screening and adherence status variables were described using frequencies and proportions.

Cronbach’s alpha coefficient was used to estimate the internal reliability of attitudes and knowledge items. Items assessing attitudes were categorised into two groups: (1) negative attitudes—representing barriers to cervical cancer screening, and (2) positive attitudes—representing enablers of cervical cancer screening. Each attitude was also dichotomised: 0 = agree (combining strongly agree and agree), and 1 = disagree (combining strongly disagree and disagree). Participants’ responses to attitudes items were described using frequencies and proportions. Attitudinal differences between adherent and non-adherent groups were tested using the Pearson Chi-square test. Mean scores and ranges for each knowledge domain were calculated for each participant. Means and standard deviation, and frequencies and proportions were used to explore participant characteristics (socio-demographic variables, cervical cancer screening accessibility-related characteristics, health status) as appropriate.

Logistic regression was used to determine factors associated with non-adherence to Eswatini cervical cancer screening recommendations. Potential factors examined included socio-demographic characteristics, cervical cancer screening accessibility-related, HIV status, cervical cancer screening knowledge (knowledge scores for the screening intervals, risk factors, benefits of screening, and meaning of screening knowledge domains), and attitudes toward cervical cancer screening. No participants reported being diagnosed with HPV, so the HPV diagnosis variable was not included in the regression analysis.

As part of model building, univariate logistic regression analysis (for socio-demographic, cervical cancer screening accessibility-related and health status characteristics), Chi-squared test of differences (for attitude items), and a less rigorous alpha level (≤ 0.25) [40, 41] were used to select variables to include in the first multivariate logistic regression model. The potential for introducing confounding bias was also considered in addition to significance. The variance inflation factor (VIF) was used to measure the amount of multicollinearity in the multivariate analysis variables. We estimated odds ratios (OR) of being classified as non-adherent with 95% confidence intervals (C.I.s) and corresponding *p*-values [42]. The significance level for the multivariate analysis was set at a 0.05 threshold. Statistics and Data (STATA) software version 16 was used to conduct all statistical analyses.

## Results

Four hundred and fifty-nine women were assessed for eligibility; 416 were eligible (reasons for ineligibility included a self-reported history of cervical cancer diagnosis and hysterectomy). Thirty-nine women declined to participate, while 377 consented to participate in the study, giving a response rate of 91%. There was no statistically significant difference between consenters’ and non-consenters’ age distributions, suggesting that there was no selection bias in the present study (Pearson’s Chi-square = 5.917, *p* = 0.44).

### Participant characteristics

Participant characteristics are shown in Table 1. The mean age of our sample was 35 years (SD = 9.6), and just below two-thirds had a secondary/ high school level of education (*n* = 229, 61%). Over half reported not living with HIV (*n* = 198, 53%), and most did not know whether or not they were living with HPV (*n* = 373, 99%).

**Table 1:** Participants’ Characteristics by Adherence Classification (*N* = 377)

Women Characteristics	All Participants	Adherent *N* = 166 (44%) n (%)	Non-adherent *N* = 211 (56%) n (%)
**Age**			
Mean (S.D., range)	35 (9.6, 25 – 59)	35 (8.2, 25–59)	35 (10.6, 25–59)
**Marital status**			
Single	160 (42)	62 (37)	98 (46)
Married/living with a partner	184 (49)	90 (54)	94 (45)
Divorced/separated/widowed	33 (9)	13 (8)	19 (9)
**Education**			
No formal education/primary school	108 (42)	56 (34)	52 (25)
Secondary/high school	229 (61)	92 (55)	137 (65)
Tertiary	40 (11)	18 (11)	22 (10)
**Self-reported HIV status**			
“HIV-positive”	179 (47)	89 (54)	90 (43)
“HIV-negative”	198 (53)	77 (46)	121 (57)
Did not know	-	-	-
**Self-reported HPV status**			
“HPV-positive”	4 (1)	-	4 (2)
“HPV-negative”	-	-	-
Did not know	373 (99)	211 (100)	162 (97)
**Availability of electricity in household**			
Yes	304 (81)	135 (81)	169 (80)
No	73 (19)	31 (19)	42 (20)
**Travel time to clinic**			
≤ 30 min	192 (51)	85 (51)	107 (51)
> 30 min	185 (49)	81 (49)	104 (49)
**Clinic visits in the past 6 months**			
More than twice	164 (44)	77 (46)	87 (41)
Twice	73 (19)	34 (20)	39 (18)
Once	80 (21)	36 (22)	44 (21)
Never	60 (16)	19 (11)	41 (19)
**Knowledge scores:** range obtained (maximum score, mean score)			
** Screening intervals**	0 – 7 (7, 2)	0 – 7 (7, 2)	0 – 5 (5, 2)
** Risk factors**	1 – 10 (10, 7)	1 – 10 (10, 7)	2 – 10 (10, 7)
** Benefits of screening**	1 – 6 (6, 4)	2 – 5 (5, 4)	1 – 6 (6, 4)
** Meaning of screening**	0 – 3 (3, 3)	0 – 3 (3, 3)	0 – 3 (3, 3)

*Abbreviations: HIV* human immunodeficiency virus, *HPV* human papillomavirus

### Adherence to Eswatini cervical cancer screening recommendations

One hundred and sixty-six (44%) women were classified as adherent to the Eswatini cervical cancer screening recommendations based on their self-reported screening participation. Details on the characteristics of the adherent and non-adherent groups are also presented in Table 1. At the time of data collection, more than half (*n* = 234, 62%) of our sample had been screened for cervical cancer at least once (i.e., “ever screened”).

### Attitudes toward cervical cancer screening

The attitude questionnaire’s internal consistency was confirmed with a Cronbach’s alpha coefficient of 0.73. Attitudinal barriers endorsed by over one-third of participants included a perceived low risk of cervical cancer (*n* = 161, 43%), and a view that screening is likely to be painful (*n* = 146, 38%). In addition, of the three enablers included in the questionnaire, two were endorsed by more than 50% of the participants: perceived ability to get to a clinic that does screening easily (*n* = 333, 88%), and being advised by a doctor/ nurse to screen (*n* = 231, 61%). Details on attitudes toward cervical cancer screening are presented in Table 2.

**Table 2:** Women’s Attitudes towards Cervical Cancer Screening by Adherence Classification (*N* = 377)

**Attitude**		**Total** **n (%)**	**Adherence Classification**		***P*-value**
			**Adherent** **n (%)**	**Non-adherent** **n (%)**	
**I think VIA screening is:**					
Too expensive	Agree	20 (5)	7 (4)	13 (6)	0.40
	Disagree	357 (95)	159 (96)	198 (94)	
Embarrassing	Agree	103 (27)	41 (25)	62 (29)	0.31
	Disagree	274 (73)	125 (75)	149 (71)	
Likely to be painful	Agree	146 (38)	34 (20)	112 (53)	< 0.001^a^
	Disagree	231 (61)	132 (80)	99 (47)	
**I am:**					
At low risk of cervical cancer	Agree	161 (43)	83 (50)	78 (37)	0.01^a^
	Disagree	216 (57)	83 (50)	133 (63)	
Not comfortable being screened by younger doctors/ nurses	Agree	81 (21)	32 (19)	49 (23)	0.35
	Disagree	296 (79)	134 (81)	162 (77)	
Not comfortable being screened by male doctors/nurses	Agree	126 (33)	55 (33)	71 (34)	0.91
	Disagree	251 (67)	111 (67)	140 (66)	
Able to get to a clinic that does screening easily	Agree	333 (88)	148 (89)	185 (88)	0.66
	Disagree	44 (12)	18 (11)	26 (12)	
Worried that screening would show that I have cervical cancer	Agree	114 (30)	57 (34)	57 (27)	0.12^a^
	Disagree	263 (70)	109 (66)	154 (73)	
Too busy to undergo VIA screening	Agree	66 (18)	35 (21)	31 (15)	
	Disagree	311 (82)	131 (79)	180 (85)	0.01^a^
**I have been:**					
Advised by a doctor/ nurse to screen	Agree	231 (61)	126 (76)	105 (50)	< 0.001^a^
	Disagree	146 (39)	40 (24)	106 (50)	
Encouraged by a friend, partner or family member to screen	Agree	120 (32)	55 (33)	65 (31)	0.46
	Disagree	256 (68)	110 (66)	146 (69)	

*Abbreviation: *VIA visual inspection with acetic acid

^a^Statistically significant at α = 0.25

### Factors associated with non-adherence to Eswatini cervical cancer screening recommendations

As part of final regression model building, we assessed for confounding effects; no confounding effects were found. The meaning of screening knowledge score had VIF = 12.41 and, after further investigation, was included in the final model based on a tolerance (1/VIF) less than 0.1 (1/VIF = 0.08). As shown in Table 3, a 1-year increase in age was associated with 1.42 times increase in the odds of being classified as non-adherent (OR = 1.42, 95% CI: 1.39, 1.45, *p* = 0.01). Single participants had 1.85 times higher odds of being classified as non-adherent compared to married participants (OR = 1.78, 95% CI: 1.05, 3.01, *p* = 0.03). Moreover, participants who agreed that screening is likely painful were more likely to be classified as non-adherent (OR = 4.43, 95% CI: 2.62, 7.46, *p* < 0.001) compared to those who disagreed that screening is likely painful. Furthermore, participants who disagreed that they were advised by a doctor/ nurse to screen were 2.49 times more likely to be classified as non-adherent (OR = 2.82, 95% CI: 1.71, 4.64, *p* < 0.001) compared to those who agreed that they were advised by a doctor/ nurse to screen.

**Table 3:** Factors Associated with Non-adherence to Screening Recommendations (*N* = 377)

Women Characteristics	Unadjusted OR (95% CI)	*p*-value	Adjusted OR (95% CI)	*p*-value
**Age**	1.01 (0.98 – 1.03)	0.12 ^a^	1.42 (1.39 – 1.45)	0.01^b^
**Marital status**				
Single	1.64 (1.05 – 2.58)	0.03^a^	1.78 (1.05 – 3.01)	0.03^b^
Married/living with a partner	R		R	
Divorced/separated/widowed	1.41 (0.66 – 3.02)	0.69	1.26 (0.51 – 3.14)	0.62
**Education**				
No formal education/ primary school	R		R	
Secondary/ high school	1.60 (1.01 – 2.54)	0.05^a^	1.56 (0.87 – 2.79)	0.13
Tertiary	0.97 (0.64 – 2.73)	0.46	1.11 (0.45 – 2.76)	0.81
**HIV status**				
“HIV-positive”	R		R	
“HIV-negative”	1.55 (1.03 – 2.34)	0.04^a^	1.04 (0.64 – 1.70)	0.86
**Availability of electricity in household**				
Yes	R		-	
No	1.08 (0.65 – 1.81)	0.76	-	-
**Travel time to clinic**				
≤ 30 min	R		-	-
> 30 min	1.02 (0.68 – 1.53)	0.92	-	-
**Clinic visits in the past 6 months**				
More than twice	R		R	
Twice	1.02 (0.58 – 1.76)	0.96	0.93 (0.49 – 1.74)	0.82
Once	1.08 (0.63 – 1.85)	0.77	1.09 (0.59 – 2.03)	0.79
Never	1.91 (1.02 – 3.57)	0.04^a^	1.10 (0.54 – 2.25)	0.79
**Knowledge**				
Screening intervals	0.76 (0.60 – 0.97)	0.03^a^	0.80 (0.50 – 1.06)	0.12
Risk factors	0.99 (0.89 – 1.12)	0.95	-	-
Benefits of screening	0.93 (0.73 – 1.17)	0.52	-	-
Meaning of screening	1.51 (1.06 – 2.16)	0.02^a^	1.73 (0.91 – 2.64)	0.19
**Attitudes**				
**Screening is likely painful**				
Agree	-	-	4.43 (2.62 – 7.46)	< 0.001^b^
Disagree	-		R	
**At low risk of getting cancer**				
Agree	-	-	0.63 (0.39 – 1.01)	0.06
Disagree	-		R	
**Screening would show cervical cancer**				
Agree	-	-	0.62 (0.37 – 1.03)	0.06
Disagree	-		R	
**Advised by a doctor/ nurse to screen**				
Agree	-		R	
Disagree	-	-	2.82 (1.71 – 4.64)	< 0.001^b^

*Abbreviations: OR* odds ratio, *HIV* human immunodeficiency virus

^a^Statistically significant at *α* = 0.25

^b^Statistically significant at *α* = 0.05

## Discussion

Encouragingly, 44% of women reported that they had been screened in line with guideline recommendations. However, a substantial proportion (56%) of women self-reported a level of screening participation that was less than recommended by guidelines. This may be related to the opportunistic screening program implemented in Eswatini [43]. Unlike in an organised screening program where women are systemically invited to screen, in an opportunistic screening program, women may be offered or request a screening test as they present to healthcare providers for various complaints [44]. Opportunistic screening tends to be less efficient, contributes to health inequalities, and achieves lower coverage [45]. For example, in Nigeria, where opportunistic screening is being implemented [46], only 3% of eligible women had ever been screened [47]. In Eswatini, the coverage of screening is low, as less than half of primary healthcare clinics provide cervical cancer screening services [48]. These limitations associated with an opportunistic screening program may contribute to low rates of adherence to screening recommendations.

In the current study, almost all (99%) women did not know whether or not they were living with HPV. This is likely because HPV-DNA testing is not routinely conducted in Eswatini [49]. Our results also show that all participants who self-reported being “HPV-positive” were classified as non-adherent to Eswatini screening recommendations. The reason for this finding is unknown; it may be difficult to draw any real conclusions from this finding given the small number participants who self-reported being “HPV-positive”. However, women may assume that being “HPV-positive” means they would definitely develop cervical cancer in the future [50] and thus not perceive the preventive benefit of adhering to screening recommendations.

While believing that VIA screening was likely to be painful was the second most common barrier, almost two-thirds of the participants believed cervical cancer screening was unlikely to be painful. In a similar study in Nigeria, participants expressed that screening was uncomfortable rather than painful [51]. The current study demonstrated that women who believed that cervical cancer screening was likely to be painful were more likely to be classified as non-adherent than women who believed screening was unlikely to be painful. Fear of pain is common among women who have never been screened before [52]. Fear may be exacerbated by rumours from close family members or friends that the screening process is painful [53]. Educational messages must allay these fears by emphasising that screening is not painful and highlighting the benefits of screening [54, 55].

Women who reported that a doctor or nurse did not advise them to screen had higher odds of being classified as non-adherent than those who reported that a doctor or nurse advised them. Previous studies indicate that healthcare providers’ recommendations may increase cervical cancer screening intention and utilisation [23, 39, 56, 57]. For example, a study in eastern Uganda reported that respondents recommended for screening by a healthcare provider were 87 times more likely to be screened for cervical cancer [56]. These findings highlight how healthcare providers’ role is critical to the success of the cervical cancer screening program.

We reported that a 1-year increase in age was associated with an increase in the odds of being classified as non-adherent. This finding aligns with the results of studies conducted in Ghana [20], South Africa [58], and Canada [59]. An increasing vulnerability to health problems as one age may underlie this finding [60, 61]. A higher burden of chronic illnesses and multiple healthcare needs among older women may result in them prioritising curative care over preventive care [62]. Also, due to time constraints, health providers may focus on managing older adults’ chronic illnesses, focusing less on preventive care such as cervical cancer screening [63]. Future studies to explore the complexities of priority setting involving cervical cancer screening (among older adults) may be necessary for Eswatini.

Additionally, the reduced availability of screening among older Swati women may explain why they were classified as non-adherent to cervical cancer screening recommendations in the current study. The Eswatini standardised cancer guidelines recommend that older women screen for cervical cancer using a Pap smear. Unfortunately, a Pap smear is currently unavailable at public primary healthcare clinics, where many women access healthcare services. Pap smear can only be accessed in regional hospitals and private clinics [43].

Consistent with previous studies [22, 39], we found that single participants were 1.85 times more likely to be classified as non-adherent compared to married participants. A systematic review of factors affecting women’s health services utilisation in developing countries suggested that demand for services is lower among single women than married women [64]. As a result, health professionals have fewer opportunities to educate and encourage single women to participate in cervical screening. Also, single women are less likely to receive emotional and financial support to screen. Existing literature has suggested a lack of emotional support, encouragement, and financial support from partners as an overlooked risk factor for non-adherence [65].

## Strengths and limitations

Unlike most studies conducted in the African setting, the current research has considered both the history of ever screening and the regularity of screening to classify participants as adherent or not adherent to screening guidelines. Our study has limitations that suggest results should be interpreted with caution. First, this study was limited to women accessing healthcare services at four primary healthcare facilities in Eswatini; thus, results may not be generalisable to women attending other clinics. In addition, adherence to cervical cancer screening recommendations was classified based on self-reported screening participation, possibly resulting in an overestimation of adherence to cervical cancer screening recommendations in Eswatini [66].

## Conclusions

While screening remains the primary option for cervical cancer prevention in Eswatini, the current study suggests that there may be high levels of non-adherence to cervical cancer screening guidelines in Eswatini. Given the observation that aging, single, perceived that screening was likely painful, and reported not being advised by a doctor/nurse to screen were more likely to be classified as non-adherent to Eswatini cervical cancer screening recommendations, we suggest that healthcare providers pay particular attention to the screening needs of these women. Inviting and addressing these women’s attitudinal barriers toward screening with health education may improve screening participation and adherence to Eswatini cervical cancer screening recommendations.

## Data Availability

The datasets generated during and/or analysed during the current study are available from the corresponding author upon reasonable request.
